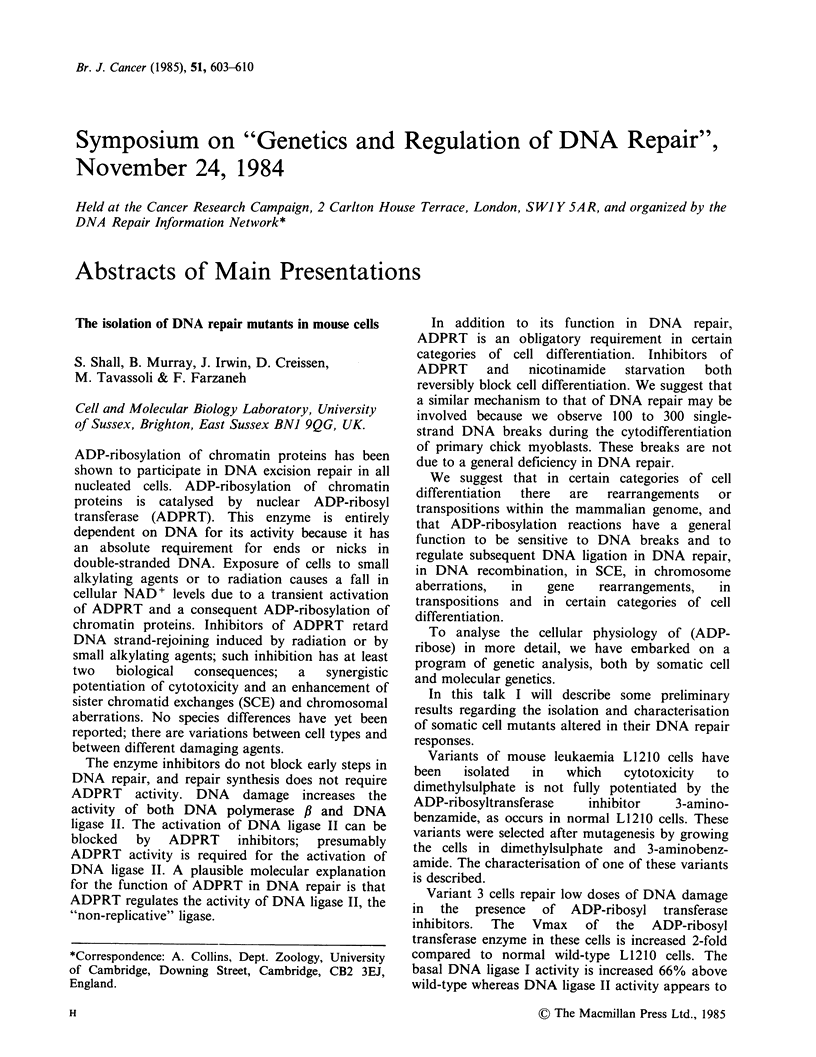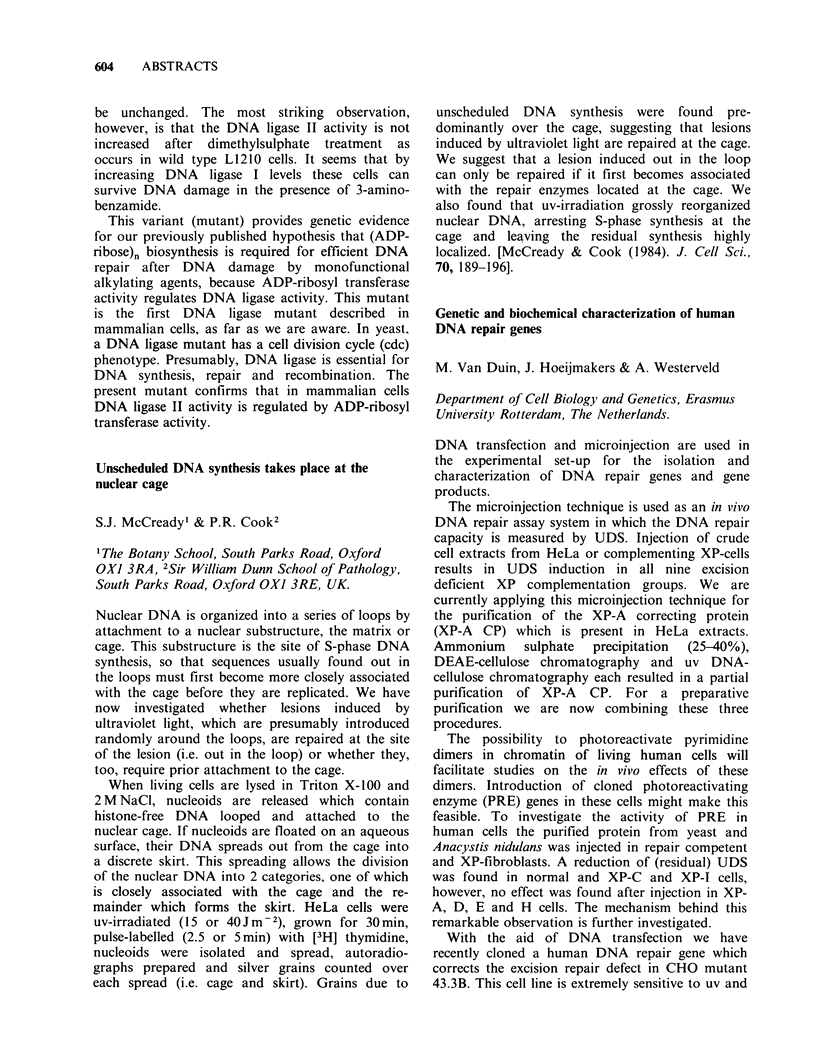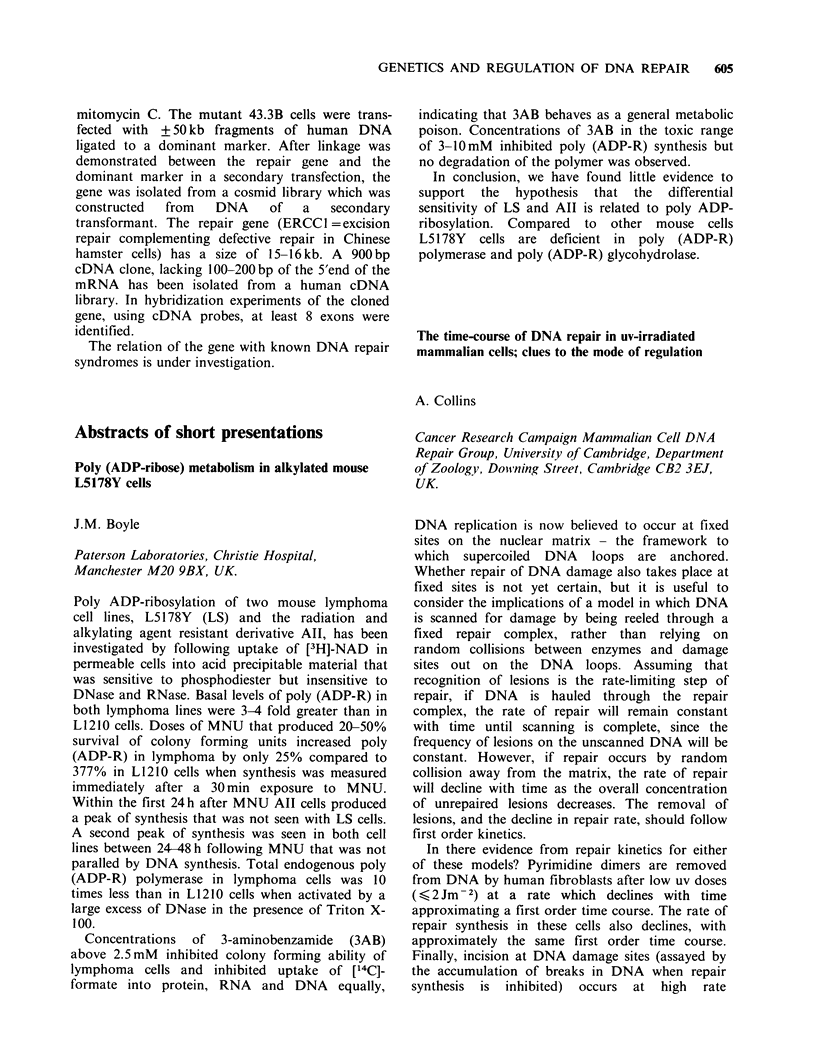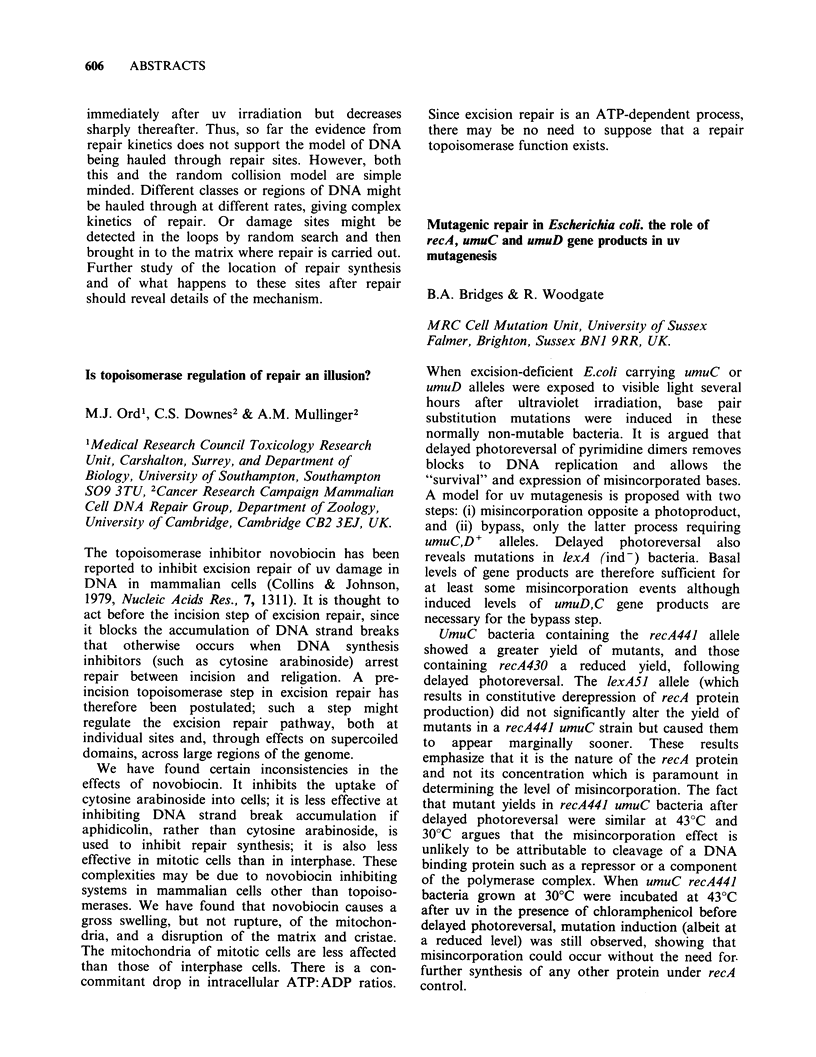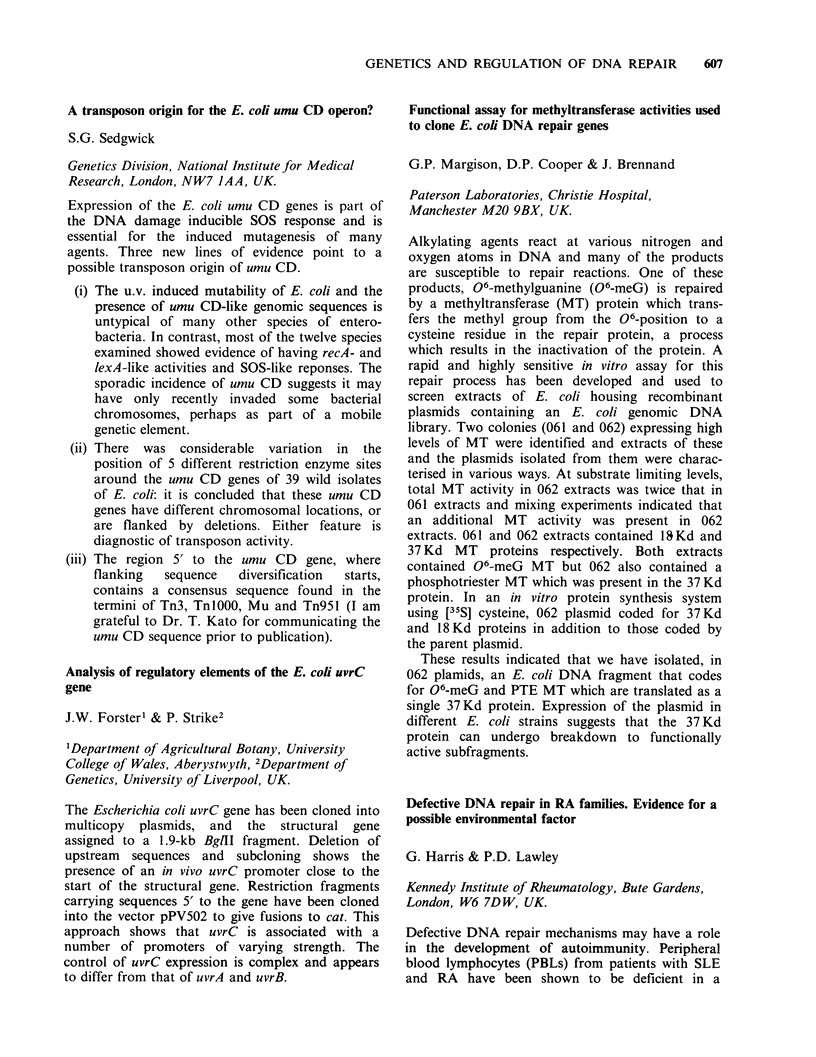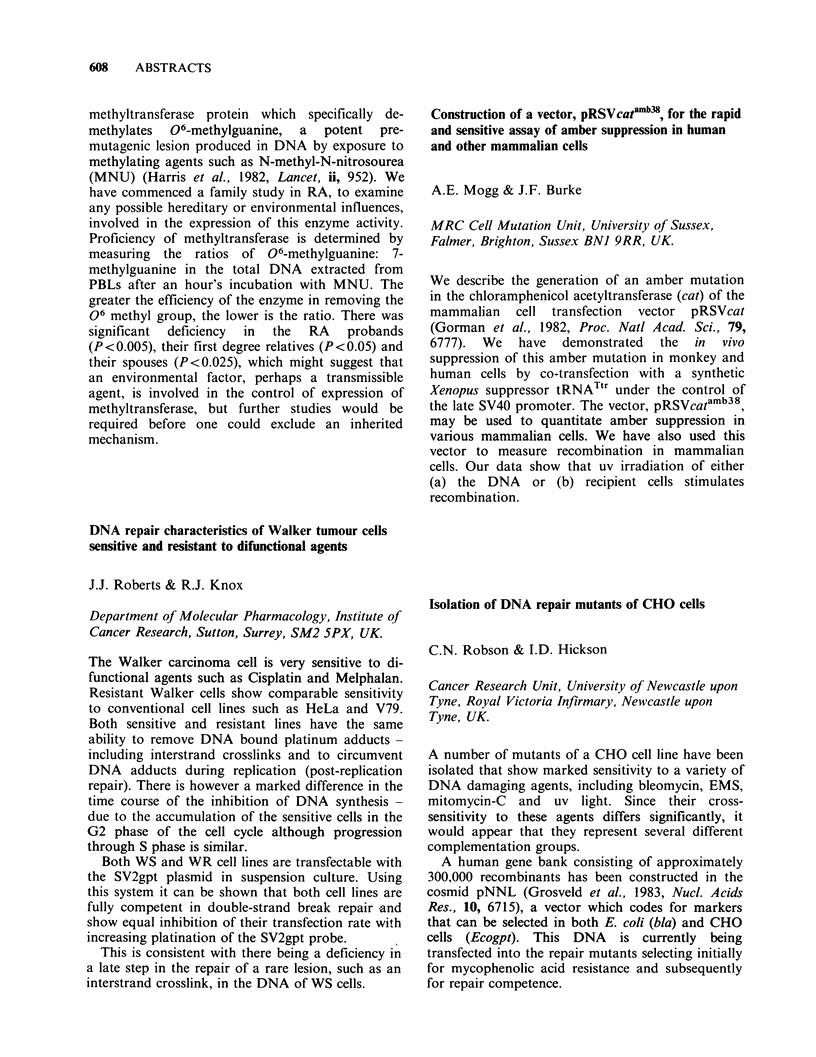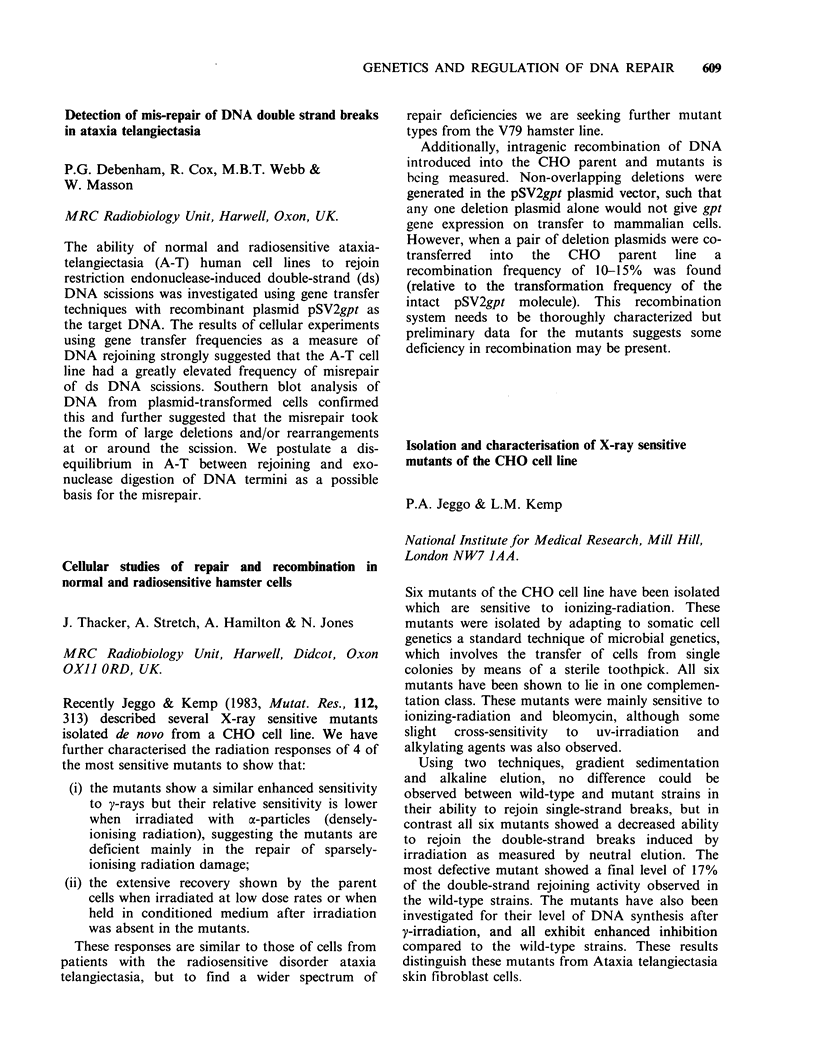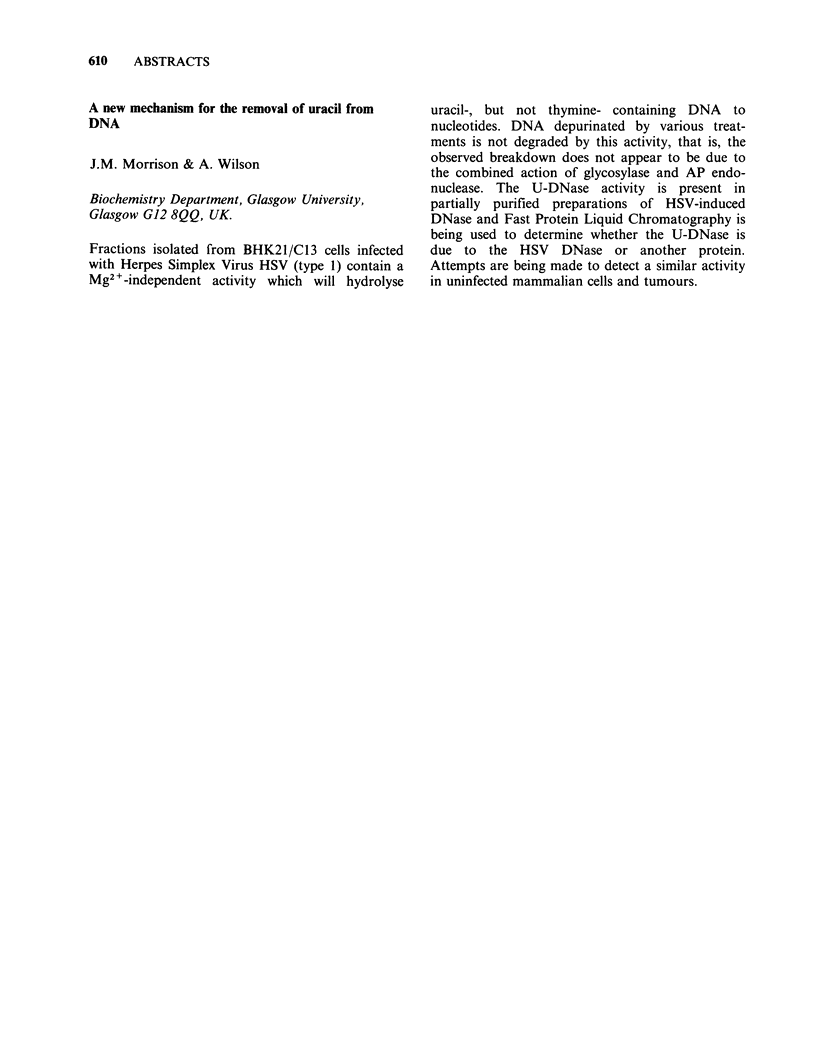# Symposium on "Genetics and regulation of DNA repair", November 24, 1984, London. Abstracts.

**DOI:** 10.1038/bjc.1985.83

**Published:** 1985-04

**Authors:** 


*Br. J. Cancer* (1985), **51**, 603–610

### Symposium on "Genetics and Regulation of DNA Repair", November 24, 1984

*Held at the Cancer Research Campaign, 2 Carlton House Terrace, London, SW1Y 5AR, and organized by the DNA Repair Information Network**

### Abstracts of Main Presentations

### The isolation of DNA repair mutants in mouse cells

S. Shall, B. Murray, J. Irwin, D. Creissen, M. Tavassoli & F. Farzaneh

*Cell and Molecular Biology Laboratory, University of Sussex, Brighton, East Sussex BN1 9QG, UK.*

ADP-ribosylation of chromatin proteins has been shown to participate in DNA excision repair in all nucleated cells. ADP-ribosylation of chromatin proteins is catalysed by nuclear ADP-ribosyl transferase (ADPRT). This enzyme is entirely dependent on DNA for its activity because it has an absolute requirement for ends or nicks in double-stranded DNA. Exposure of cells to small alkylating agents or to radiation causes a fall in cellular $NAD^+$ levels due to a transient activation of ADPRT and a consequent ADP-ribosylation of chromatin proteins. Inhibitors of ADPRT retard DNA strand-rejoining induced by radiation or by small alkylating agents; such inhibition has at least two biological consequences; a synergistic potentiation of cytotoxicity and an enhancement of sister chromatid exchanges (SCE) and chromosomal aberrations. No species differences have yet been reported; there are variations between cell types and between different damaging agents.

The enzyme inhibitors do not block early steps in DNA repair, and repair synthesis does not require ADPRT activity. DNA damage increases the activity of both DNA polymerase $\beta$ and DNA ligase II. The activation of DNA ligase II can be blocked by ADPRT inhibitors; presumably ADPRT activity is required for the activation of DNA ligase II. A plausible molecular explanation for the function of ADPRT in DNA repair is that ADPRT regulates the activity of DNA ligase II, the "non-replicative" ligase.

In addition to its function in DNA repair, ADPRT is an obligatory requirement in certain categories of cell differentiation. Inhibitors of ADPRT and nicotinamide starvation both reversibly block cell differentiation. We suggest that a similar mechanism to that of DNA repair may be involved because we observe 100 to 300 single-strand DNA breaks during the cytodifferentiation of primary chick myoblasts. These breaks are not due to a general deficiency in DNA repair.

We suggest that in certain categories of cell differentiation there are rearrangements or transpositions within the mammalian genome, and that ADP-ribosylation reactions have a general function to be sensitive to DNA breaks and to regulate subsequent DNA ligation in DNA repair, in DNA recombination, in SCE, in chromosome aberrations, in gene rearrangements, in transpositions and in certain categories of cell differentiation.

To analyse the cellular physiology of (ADP-ribose) in more detail, we have embarked on a program of genetic analysis, both by somatic cell and molecular genetics.

In this talk I will describe some preliminary results regarding the isolation and characterisation of somatic cell mutants altered in their DNA repair responses.

Variants of mouse leukaemia L1210 cells have been isolated in which cytotoxicity to dimethylsulphate is not fully potentiated by the ADP-ribosyltransferase inhibitor 3-amino-benzamide, as occurs in normal L1210 cells. These variants were selected after mutagenesis by growing the cells in dimethylsulphate and 3-aminobenz-amide. The characterisation of one of these variants is described.

Variant 3 cells repair low doses of DNA damage in the presence of ADP-ribosyl transferase inhibitors. The Vmax of the ADP-ribosyl transferase enzyme in these cells is increased 2-fold compared to normal wild-type L1210 cells. The basal DNA ligase I activity is increased 66% above wild-type whereas DNA ligase II activity appears to

*Correspondence: A. Collins, Dept. Zoology, University of Cambridge, Downing Street, Cambridge, CB2 3EJ, England.

© The Macmillan Press Ltd., 1985

be unchanged. The most striking observation, however, is that the DNA ligase II activity is not increased after dimethylsulphate treatment as occurs in wild type L1210 cells. It seems that by increasing DNA ligase I levels these cells can survive DNA damage in the presence of 3-aminobenzamide.

This variant (mutant) provides genetic evidence for our previously published hypothesis that (ADP-ribose)$_n$ biosynthesis is required for efficient DNA repair after DNA damage by monofunctional alkylating agents, because ADP-ribosyl transferase activity regulates DNA ligase activity. This mutant is the first DNA ligase mutant described in mammalian cells, as far as we are aware. In yeast, a DNA ligase mutant has a cell division cycle (cdc) phenotype. Presumably, DNA ligase is essential for DNA synthesis, repair and recombination. The present mutant confirms that in mammalian cells DNA ligase II activity is regulated by ADP-ribosyl transferase activity.

### Unscheduled DNA synthesis takes place at the nuclear cage

S.J. McCready[1] & P.R. Cook[2]

[1]*The Botany School, South Parks Road, Oxford OX1 3RA,* [2]*Sir William Dunn School of Pathology, South Parks Road, Oxford OX1 3RE, UK.*

Nuclear DNA is organized into a series of loops by attachment to a nuclear substructure, the matrix or cage. This substructure is the site of S-phase DNA synthesis, so that sequences usually found out in the loops must first become more closely associated with the cage before they are replicated. We have now investigated whether lesions induced by ultraviolet light, which are presumably introduced randomly around the loops, are repaired at the site of the lesion (i.e. out in the loop) or whether they, too, require prior attachment to the cage.

When living cells are lysed in Triton X-100 and 2 M NaCl, nucleoids are released which contain histone-free DNA looped and attached to the nuclear cage. If nucleoids are floated on an aqueous surface, their DNA spreads out from the cage into a discrete skirt. This spreading allows the division of the nuclear DNA into 2 categories, one of which is closely associated with the cage and the remainder which forms the skirt. HeLa cells were uv-irradiated (15 or 40 J m$^{-2}$), grown for 30 min, pulse-labelled (2.5 or 5 min) with [$^3$H] thymidine, nucleoids were isolated and spread, autoradiographs prepared and silver grains counted over each spread (i.e. cage and skirt). Grains due to unscheduled DNA synthesis were found predominantly over the cage, suggesting that lesions induced by ultraviolet light are repaired at the cage. We suggest that a lesion induced out in the loop can only be repaired if it first becomes associated with the repair enzymes located at the cage. We also found that uv-irradiation grossly reorganized nuclear DNA, arresting S-phase synthesis at the cage and leaving the residual synthesis highly localized. [McCready & Cook (1984). *J. Cell Sci.*, **70,** 189–196].

### Genetic and biochemical characterization of human DNA repair genes

M. Van Duin, J. Hoeijmakers & A. Westerveld

*Department of Cell Biology and Genetics, Erasmus University Rotterdam, The Netherlands.*

DNA transfection and microinjection are used in the experimental set-up for the isolation and characterization of DNA repair genes and gene products.

The microinjection technique is used as an *in vivo* DNA repair assay system in which the DNA repair capacity is measured by UDS. Injection of crude cell extracts from HeLa or complementing XP-cells results in UDS induction in all nine excision deficient XP complementation groups. We are currently applying this microinjection technique for the purification of the XP-A correcting protein (XP-A CP) which is present in HeLa extracts. Ammonium sulphate precipitation (25–40%), DEAE-cellulose chromatography and uv DNA-cellulose chromatography each resulted in a partial purification of XP-A CP. For a preparative purification we are now combining these three procedures.

The possibility to photoreactivate pyrimidine dimers in chromatin of living human cells will facilitate studies on the *in vivo* effects of these dimers. Introduction of cloned photoreactivating enzyme (PRE) genes in these cells might make this feasible. To investigate the activity of PRE in human cells the purified protein from yeast and *Anacystis nidulans* was injected in repair competent and XP-fibroblasts. A reduction of (residual) UDS was found in normal and XP-C and XP-I cells, however, no effect was found after injection in XP-A, D, E and H cells. The mechanism behind this remarkable observation is further investigated.

With the aid of DNA transfection we have recently cloned a human DNA repair gene which corrects the excision repair defect in CHO mutant 43.3B. This cell line is extremely sensitive to uv and mitomycin C. The mutant 43.3B cells were transfected with ±50 kb fragments of human DNA ligated to a dominant marker. After linkage was demonstrated between the repair gene and the dominant marker in a secondary transfection, the gene was isolated from a cosmid library which was constructed from DNA of a secondary transformant. The repair gene (ERCC1 = excision repair complementing defective repair in Chinese hamster cells) has a size of 15–16 kb. A 900 bp cDNA clone, lacking 100–200 bp of the 5′end of the mRNA has been isolated from a human cDNA library. In hybridization experiments of the cloned gene, using cDNA probes, at least 8 exons were identified.

The relation of the gene with known DNA repair syndromes is under investigation.

### Abstracts of short presentations

### Poly (ADP-ribose) metabolism in alkylated mouse L5178Y cells

J.M. Boyle

*Paterson Laboratories, Christie Hospital, Manchester M20 9BX, UK.*

Poly ADP-ribosylation of two mouse lymphoma cell lines, L5178Y (LS) and the radiation and alkylating agent resistant derivative AII, has been investigated by following uptake of [$^3$H]-NAD in permeable cells into acid precipitable material that was sensitive to phosphodiester but insensitive to DNase and RNase. Basal levels of poly (ADP-R) in both lymphoma lines were 3–4 fold greater than in L1210 cells. Doses of MNU that produced 20–50% survival of colony forming units increased poly (ADP-R) in lymphoma by only 25% compared to 377% in L1210 cells when synthesis was measured immediately after a 30 min exposure to MNU. Within the first 24 h after MNU AII cells produced a peak of synthesis that was not seen with LS cells. A second peak of synthesis was seen in both cell lines between 24–48 h following MNU that was not paralled by DNA synthesis. Total endogenous poly (ADP-R) polymerase in lymphoma cells was 10 times less than in L1210 cells when activated by a large excess of DNase in the presence of Triton X-100.

Concentrations of 3-aminobenzamide (3AB) above 2.5 mM inhibited colony forming ability of lymphoma cells and inhibited uptake of [$^{14}$C]-formate into protein, RNA and DNA equally, indicating that 3AB behaves as a general metabolic poison. Concentrations of 3AB in the toxic range of 3–10 mM inhibited poly (ADP-R) synthesis but no degradation of the polymer was observed.

In conclusion, we have found little evidence to support the hypothesis that the differential sensitivity of LS and AII is related to poly ADP-ribosylation. Compared to other mouse cells L5178Y cells are deficient in poly (ADP-R) polymerase and poly (ADP-R) glycohydrolase.

### The time-course of DNA repair in uv-irradiated mammalian cells; clues to the mode of regulation

A. Collins

*Cancer Research Campaign Mammalian Cell DNA Repair Group, University of Cambridge, Department of Zoology, Downing Street, Cambridge CB2 3EJ, UK.*

DNA replication is now believed to occur at fixed sites on the nuclear matrix – the framework to which supercoiled DNA loops are anchored. Whether repair of DNA damage also takes place at fixed sites is not yet certain, but it is useful to consider the implications of a model in which DNA is scanned for damage by being reeled through a fixed repair complex, rather than relying on random collisions between enzymes and damage sites out on the DNA loops. Assuming that recognition of lesions is the rate-limiting step of repair, if DNA is hauled through the repair complex, the rate of repair will remain constant with time until scanning is complete, since the frequency of lesions on the unscanned DNA will be constant. However, if repair occurs by random collision away from the matrix, the rate of repair will decline with time as the overall concentration of unrepaired lesions decreases. The removal of lesions, and the decline in repair rate, should follow first order kinetics.

In there evidence from repair kinetics for either of these models? Pyrimidine dimers are removed from DNA by human fibroblasts after low uv doses ($\leqslant 2\,\mathrm{Jm}^{-2}$) at a rate which declines with time approximating a first order time course. The rate of repair synthesis in these cells also declines, with approximately the same first order time course. Finally, incision at DNA damage sites (assayed by the accumulation of breaks in DNA when repair synthesis is inhibited) occurs at high rate immediately after uv irradiation but decreases sharply thereafter. Thus, so far the evidence from repair kinetics does not support the model of DNA being hauled through repair sites. However, both this and the random collision model are simple minded. Different classes or regions of DNA might be hauled through at different rates, giving complex kinetics of repair. Or damage sites might be detected in the loops by random search and then brought in to the matrix where repair is carried out. Further study of the location of repair synthesis and of what happens to these sites after repair should reveal details of the mechanism.

**Is topoisomerase regulation of repair an illusion?**

M.J. Ord[1], C.S. Downes[2] & A.M. Mullinger[2]

[1]*Medical Research Council Toxicology Research Unit, Carshalton, Surrey, and Department of Biology, University of Southampton, Southampton SO9 3TU,* [2]*Cancer Research Campaign Mammalian Cell DNA Repair Group, Department of Zoology, University of Cambridge, Cambridge CB2 3EJ, UK.*

The topoisomerase inhibitor novobiocin has been reported to inhibit excision repair of uv damage in DNA in mammalian cells (Collins & Johnson, 1979, *Nucleic Acids Res.*, **7**, 1311). It is thought to act before the incision step of excision repair, since it blocks the accumulation of DNA strand breaks that otherwise occurs when DNA synthesis inhibitors (such as cytosine arabinoside) arrest repair between incision and religation. A pre-incision topoisomerase step in excision repair has therefore been postulated; such a step might regulate the excision repair pathway, both at individual sites and, through effects on supercoiled domains, across large regions of the genome.

We have found certain inconsistencies in the effects of novobiocin. It inhibits the uptake of cytosine arabinoside into cells; it is less effective at inhibiting DNA strand break accumulation if aphidicolin, rather than cytosine arabinoside, is used to inhibit repair synthesis; it is also less effective in mitotic cells than in interphase. These complexities may be due to novobiocin inhibiting systems in mammalian cells other than topoisomerases. We have found that novobiocin causes a gross swelling, but not rupture, of the mitochondria, and a disruption of the matrix and cristae. The mitochondria of mitotic cells are less affected than those of interphase cells. There is a concommitant drop in intracellular ATP:ADP ratios. Since excision repair is an ATP-dependent process, there may be no need to suppose that a repair topoisomerase function exists.

**Mutagenic repair in *Escherichia coli.* the role of *recA*, *umuC* and *umuD* gene products in uv mutagenesis**

B.A. Bridges & R. Woodgate

*MRC Cell Mutation Unit, University of Sussex Falmer, Brighton, Sussex BN1 9RR, UK.*

When excision-deficient *E.coli* carrying *umuC* or *umuD* alleles were exposed to visible light several hours after ultraviolet irradiation, base pair substitution mutations were induced in these normally non-mutable bacteria. It is argued that delayed photoreversal of pyrimidine dimers removes blocks to DNA replication and allows the "survival" and expression of misincorporated bases. A model for uv mutagenesis is proposed with two steps: (i) misincorporation opposite a photoproduct, and (ii) bypass, only the latter process requiring *umuC,D*$^+$ alleles. Delayed photoreversal also reveals mutations in *lexA* (ind$^-$) bacteria. Basal levels of gene products are therefore sufficient for at least some misincorporation events although induced levels of *umuD,C* gene products are necessary for the bypass step.

*UmuC* bacteria containing the *recA441* allele showed a greater yield of mutants, and those containing *recA430* a reduced yield, following delayed photoreversal. The *lexA51* allele (which results in constitutive derepression of *recA* protein production) did not significantly alter the yield of mutants in a *recA441 umuC* strain but caused them to appear marginally sooner. These results emphasize that it is the nature of the *recA* protein and not its concentration which is paramount in determining the level of misincorporation. The fact that mutant yields in *recA441 umuC* bacteria after delayed photoreversal were similar at 43°C and 30°C argues that the misincorporation effect is unlikely to be attributable to cleavage of a DNA binding protein such as a repressor or a component of the polymerase complex. When *umuC recA441* bacteria grown at 30°C were incubated at 43°C after uv in the presence of chloramphenicol before delayed photoreversal, mutation induction (albeit at a reduced level) was still observed, showing that misincorporation could occur without the need for further synthesis of any other protein under *recA* control.

### A transposon origin for the *E. coli umu* CD operon?

S.G. Sedgwick

*Genetics Division, National Institute for Medical Research, London, NW7 1AA, UK.*

Expression of the *E. coli umu* CD genes is part of the DNA damage inducible SOS response and is essential for the induced mutagenesis of many agents. Three new lines of evidence point to a possible transposon origin of *umu* CD.

(i) The u.v. induced mutability of *E. coli* and the presence of *umu* CD-like genomic sequences is untypical of many other species of enterobacteria. In contrast, most of the twelve species examined showed evidence of having *recA*- and *lexA*-like activities and SOS-like reponses. The sporadic incidence of *umu* CD suggests it may have only recently invaded some bacterial chromosomes, perhaps as part of a mobile genetic element.

(ii) There was considerable variation in the position of 5 different restriction enzyme sites around the *umu* CD genes of 39 wild isolates of *E. coli*: it is concluded that these *umu* CD genes have different chromosomal locations, or are flanked by deletions. Either feature is diagnostic of transposon activity.

(iii) The region 5′ to the *umu* CD gene, where flanking sequence diversification starts, contains a consensus sequence found in the termini of Tn3, Tn1000, Mu and Tn951 (I am grateful to Dr. T. Kato for communicating the *umu* CD sequence prior to publication).

### Analysis of regulatory elements of the *E. coli uvrC* gene

J.W. Forster[1] & P. Strike[2]

[1]*Department of Agricultural Botany, University College of Wales, Aberystwyth,* [2]*Department of Genetics, University of Liverpool, UK.*

The *Escherichia coli uvrC* gene has been cloned into multicopy plasmids, and the structural gene assigned to a 1.9-kb *Bgl*II fragment. Deletion of upstream sequences and subcloning shows the presence of an *in vivo uvrC* promoter close to the start of the structural gene. Restriction fragments carrying sequences 5′ to the gene have been cloned into the vector pPV502 to give fusions to *cat*. This approach shows that *uvrC* is associated with a number of promoters of varying strength. The control of *uvrC* expression is complex and appears to differ from that of *uvrA* and *uvrB*.

### Functional assay for methyltransferase activities used to clone *E. coli* DNA repair genes

G.P. Margison, D.P. Cooper & J. Brennand

*Paterson Laboratories, Christie Hospital, Manchester M20 9BX, UK.*

Alkylating agents react at various nitrogen and oxygen atoms in DNA and many of the products are susceptible to repair reactions. One of these products, $O^6$-methylguanine ($O^6$-meG) is repaired by a methyltransferase (MT) protein which transfers the methyl group from the $O^6$-position to a cysteine residue in the repair protein, a process which results in the inactivation of the protein. A rapid and highly sensitive *in vitro* assay for this repair process has been developed and used to screen extracts of *E. coli* housing recombinant plasmids containing an *E. coli* genomic DNA library. Two colonies (061 and 062) expressing high levels of MT were identified and extracts of these and the plasmids isolated from them were characterised in various ways. At substrate limiting levels, total MT activity in 062 extracts was twice that in 061 extracts and mixing experiments indicated that an additional MT activity was present in 062 extracts. 061 and 062 extracts contained 18 Kd and 37 Kd MT proteins respectively. Both extracts contained $O^6$-meG MT but 062 also contained a phosphotriester MT which was present in the 37 Kd protein. In an *in vitro* protein synthesis system using [$^{35}$S] cysteine, 062 plasmid coded for 37 Kd and 18 Kd proteins in addition to those coded by the parent plasmid.

These results indicated that we have isolated, in 062 plamids, an *E. coli* DNA fragment that codes for $O^6$-meG and PTE MT which are translated as a single 37 Kd protein. Expression of the plasmid in different *E. coli* strains suggests that the 37 Kd protein can undergo breakdown to functionally active subfragments.

### Defective DNA repair in RA families. Evidence for a possible environmental factor

G. Harris & P.D. Lawley

*Kennedy Institute of Rheumatology, Bute Gardens, London, W6 7DW, UK.*

Defective DNA repair mechanisms may have a role in the development of autoimmunity. Peripheral blood lymphocytes (PBLs) from patients with SLE and RA have been shown to be deficient in a methyltransferase protein which specifically demethylates $O^6$-methylguanine, a potent premutagenic lesion produced in DNA by exposure to methylating agents such as N-methyl-N-nitrosourea (MNU) (Harris *et al.*, 1982, *Lancet*, **ii,** 952). We have commenced a family study in RA, to examine any possible hereditary or environmental influences, involved in the expression of this enzyme activity. Proficiency of methyltransferase is determined by measuring the ratios of $O^6$-methylguanine: 7-methylguanine in the total DNA extracted from PBLs after an hour's incubation with MNU. The greater the efficiency of the enzyme in removing the $O^6$ methyl group, the lower is the ratio. There was significant deficiency in the RA probands ($P<0.005$), their first degree relatives ($P<0.05$) and their spouses ($P<0.025$), which might suggest that an environmental factor, perhaps a transmissible agent, is involved in the control of expression of methyltransferase, but further studies would be required before one could exclude an inherited mechanism.

**DNA repair characteristics of Walker tumour cells sensitive and resistant to difunctional agents**

J.J. Roberts & R.J. Knox

*Department of Molecular Pharmacology, Institute of Cancer Research, Sutton, Surrey, SM2 5PX, UK.*

The Walker carcinoma cell is very sensitive to difunctional agents such as Cisplatin and Melphalan. Resistant Walker cells show comparable sensitivity to conventional cell lines such as HeLa and V79. Both sensitive and resistant lines have the same ability to remove DNA bound platinum adducts – including interstrand crosslinks and to circumvent DNA adducts during replication (post-replication repair). There is however a marked difference in the time course of the inhibition of DNA synthesis – due to the accumulation of the sensitive cells in the G2 phase of the cell cycle although progression through S phase is similar.

Both WS and WR cell lines are transfectable with the SV2gpt plasmid in suspension culture. Using this system it can be shown that both cell lines are fully competent in double-strand break repair and show equal inhibition of their transfection rate with increasing platination of the SV2gpt probe.

This is consistent with there being a deficiency in a late step in the repair of a rare lesion, such as an interstrand crosslink, in the DNA of WS cells.

**Construction of a vector, pRSV*cat*$^{amb38}$, for the rapid and sensitive assay of amber suppression in human and other mammalian cells**

A.E. Mogg & J.F. Burke

*MRC Cell Mutation Unit, University of Sussex, Falmer, Brighton, Sussex BN1 9RR, UK.*

We describe the generation of an amber mutation in the chloramphenicol acetyltransferase (*cat*) of the mammalian cell transfection vector pRSV*cat* (Gorman *et al.*, 1982, *Proc. Natl Acad. Sci.*, **79,** 6777). We have demonstrated the *in vivo* suppression of this amber mutation in monkey and human cells by co-transfection with a synthetic *Xenopus* suppressor tRNA$^{Ttr}$ under the control of the late SV40 promoter. The vector, pRSV*cat*$^{amb38}$, may be used to quantitate amber suppression in various mammalian cells. We have also used this vector to measure recombination in mammalian cells. Our data show that uv irradiation of either (a) the DNA or (b) recipient cells stimulates recombination.

**Isolation of DNA repair mutants of CHO cells**

C.N. Robson & I.D. Hickson

*Cancer Research Unit, University of Newcastle upon Tyne, Royal Victoria Infirmary, Newcastle upon Tyne, UK.*

A number of mutants of a CHO cell line have been isolated that show marked sensitivity to a variety of DNA damaging agents, including bleomycin, EMS, mitomycin-C and uv light. Since their cross-sensitivity to these agents differs significantly, it would appear that they represent several different complementation groups.

A human gene bank consisting of approximately 300,000 recombinants has been constructed in the cosmid pNNL (Grosveld *et al.*, 1983, *Nucl. Acids Res.*, **10,** 6715), a vector which codes for markers that can be selected in both *E. coli* (*bla*) and CHO cells (*Ecogpt*). This DNA is currently being transfected into the repair mutants selecting initially for mycophenolic acid resistance and subsequently for repair competence.

### Detection of mis-repair of DNA double strand breaks in ataxia telangiectasia

P.G. Debenham, R. Cox, M.B.T. Webb & W. Masson

*MRC Radiobiology Unit, Harwell, Oxon, UK.*

The ability of normal and radiosensitive ataxia-telangiectasia (A-T) human cell lines to rejoin restriction endonuclease-induced double-strand (ds) DNA scissions was investigated using gene transfer techniques with recombinant plasmid pSV2*gpt* as the target DNA. The results of cellular experiments using gene transfer frequencies as a measure of DNA rejoining strongly suggested that the A-T cell line had a greatly elevated frequency of misrepair of ds DNA scissions. Southern blot analysis of DNA from plasmid-transformed cells confirmed this and further suggested that the misrepair took the form of large deletions and/or rearrangements at or around the scission. We postulate a dis-equilibrium in A-T between rejoining and exo-nuclease digestion of DNA termini as a possible basis for the misrepair.

### Cellular studies of repair and recombination in normal and radiosensitive hamster cells

J. Thacker, A. Stretch, A. Hamilton & N. Jones

*MRC Radiobiology Unit, Harwell, Didcot, Oxon OX11 0RD, UK.*

Recently Jeggo & Kemp (1983, *Mutat. Res.*, **112**, 313) described several X-ray sensitive mutants isolated *de novo* from a CHO cell line. We have further characterised the radiation responses of 4 of the most sensitive mutants to show that:

(i) the mutants show a similar enhanced sensitivity to $\gamma$-rays but their relative sensitivity is lower when irradiated with $\alpha$-particles (densely-ionising radiation), suggesting the mutants are deficient mainly in the repair of sparsely-ionising radiation damage;

(ii) the extensive recovery shown by the parent cells when irradiated at low dose rates or when held in conditioned medium after irradiation was absent in the mutants.

These responses are similar to those of cells from patients with the radiosensitive disorder ataxia telangiectasia, but to find a wider spectrum of repair deficiencies we are seeking further mutant types from the V79 hamster line.

Additionally, intragenic recombination of DNA introduced into the CHO parent and mutants is being measured. Non-overlapping deletions were generated in the pSV2*gpt* plasmid vector, such that any one deletion plasmid alone would not give *gpt* gene expression on transfer to mammalian cells. However, when a pair of deletion plasmids were co-transferred into the CHO parent line a recombination frequency of 10–15% was found (relative to the transformation frequency of the intact pSV2*gpt* molecule). This recombination system needs to be thoroughly characterized but preliminary data for the mutants suggests some deficiency in recombination may be present.

### Isolation and characterisation of X-ray sensitive mutants of the CHO cell line

P.A. Jeggo & L.M. Kemp

*National Institute for Medical Research, Mill Hill, London NW7 1AA.*

Six mutants of the CHO cell line have been isolated which are sensitive to ionizing-radiation. These mutants were isolated by adapting to somatic cell genetics a standard technique of microbial genetics, which involves the transfer of cells from single colonies by means of a sterile toothpick. All six mutants have been shown to lie in one complementation class. These mutants were mainly sensitive to ionizing-radiation and bleomycin, although some slight cross-sensitivity to uv-irradiation and alkylating agents was also observed.

Using two techniques, gradient sedimentation and alkaline elution, no difference could be observed between wild-type and mutant strains in their ability to rejoin single-strand breaks, but in contrast all six mutants showed a decreased ability to rejoin the double-strand breaks induced by irradiation as measured by neutral elution. The most defective mutant showed a final level of 17% of the double-strand rejoining activity observed in the wild-type strains. The mutants have also been investigated for their level of DNA synthesis after $\gamma$-irradiation, and all exhibit enhanced inhibition compared to the wild-type strains. These results distinguish these mutants from Ataxia telangiectasia skin fibroblast cells.

### A new mechanism for the removal of uracil from DNA

J.M. Morrison & A. Wilson

*Biochemistry Department, Glasgow University, Glasgow G12 8QQ, UK.*

Fractions isolated from BHK21/C13 cells infected with Herpes Simplex Virus HSV (type 1) contain a $Mg^{2+}$-independent activity which will hydrolyse uracil-, but not thymine- containing DNA to nucleotides. DNA depurinated by various treatments is not degraded by this activity, that is, the observed breakdown does not appear to be due to the combined action of glycosylase and AP endonuclease. The U-DNase activity is present in partially purified preparations of HSV-induced DNase and Fast Protein Liquid Chromatography is being used to determine whether the U-DNase is due to the HSV DNase or another protein. Attempts are being made to detect a similar activity in uninfected mammalian cells and tumours.